# Psychological dynamics among Chinese and British negotiators and translators: A text-analytical study of translation manipulation in the English - Chinese translation of the Treaty of Nanking

**DOI:** 10.1371/journal.pone.0326356

**Published:** 2025-06-18

**Authors:** Manli Li, Xujun Tian

**Affiliations:** 1 Faculty of Humanities and Social Sciences, School of International Studies, Harbin Institute of Technology, Harbin, China; 2 School of Foreign Languages, Shanghai Lixin University of Accounting and Finance, Shanghai, China; Universidade Federal do Tocantins, BRAZIL

## Abstract

This paper examines translation manipulation strategies employed by Chinese and British negotiators and translators in the English Chinese translation of the Treaty of Nanking (1842) and explores the psychological dynamics behind these practices. From the perspective of Lefevere’s translation manipulation theory, the study uncovers how translation served as a tool to mediate power asymmetries and cultural differences between the two parties through a detailed text-analytical approach. The findings highlight deliberate textual manipulations, including rhetorical adjustments omissions, and additions, which reflect the translators’ dual role as mediators and ideological agents. For instance, the Chinese text consistently elevated the Qing emperor’s authority through honorific terms and omitted critical language, aligning the treaty with Qing norms while softening its unfavorable terms. Conversely, the British version emphasized precision and claims of justice, aligning with imperial objectives. Additions in the Chinese version, such as references to imperial approval and justifications for concessions, illustrate attempts to preserve Qing dignity and promote acceptance of the treaty. Overall, the study demonstrates how psychological pressures and power dynamics shaped translation outcomes, offering new insights into translation manipulation, cultural mediation, and the role of translators in constructing historical narratives.

## Introduction

The Treaty of Nanking, signed in 1842, marked a watershed moment in modern Chinese history, symbolizing the beginning of a series of unequal treaties that eroded Chinese sovereignty and reshaped China’s foreign relations [1–4]. The treaty imposed severe terms on China, including the cession of Hong Kong to Britain, the opening of five treaty ports to British trade, and the payment of substantial indemnities. Beyond its material consequences, it also set a precedent for the diplomatic and cultural subjugation of China, as reflected in the unequal power dynamics embedded in its negotiation and translation. While the treaty’s substantive inequalities have been extensively studied, its linguistic and formal inequalities – particularly in the Chinese version – have received comparatively less attention. These disparities reveal a complex interplay of political, cultural, and psychological forces that shaped the treaty’s negotiation and textual outcomes.

Translation played a pivotal role in the treaty’s negotiation, serving as the primary medium through which terms were communicated and formalized. The translation process was notably asymmetrical, with the British side maintaining complete control over both drafting and translation [1–5]. Key British translators, including John Robert Morrison, Karl Friedrich August Gutzlaff, and Sir Robert Thom, were not merely linguistic intermediaries but active participants in shaping the treaty’s content and presentation. These individuals, all proficient in Chinese language and experienced in Chinese affairs, strategically managed both the initial drafting and final translation processes. In stark contrast, the Qing negotiation team lacked any officials with English language proficiency or translation expertise, creating a significant power imbalance in the negotiation process. According to historical records [6], the British side initiated the proceedings by presenting demands in English, which their interpreters then translated and read aloud in Chinese. The subsequent negotiation process, including the drafting of preliminary agreements and final treaty text, remained firmly under British control. This arrangement created opportunities for strategic manipulation of the treaty’s language and meaning, particularly in its Chinese version.

Existing studies have approached the Treaty of Nanking from various perspectives. Scholars have provided detailed historical documentation [1–2], while Hu and Jia have examined the politics of translation and translators’ mediatory roles [7]. Recent studies have highlighted how linguistic and structural features in the Chinese version were manipulated to preserve imperial authority while obscuring the treaty’s coercive nature [4–5]. However, most existing research has focused primarily on linguistic or historical aspects, leaving the psychological dimensions of translation manipulation and negotiation largely unexplored.

This study introduces an innovative interdisciplinary approach by integrating translation studies with psychological frameworks and historical analysis. It examines how psychological factors influenced both British and Chinese negotiators and translators, exploring the emotional and strategic motivations that shaped their decisions. By analyzing translation manipulation through this multifaceted lens, the research reveals how British translators strategically adapted the treaty’s language to achieve their objectives while maintaining diplomatic appearances, and how Chinese officials responded to these linguistic and psychological pressures.

Through comprehensive analysis, the study contributes to our understanding of how translation served as both a tool and a symbol of power in nineteenth-century diplomatic relations. It demonstrates how psychological dynamics influenced translation choices and negotiation strategies, offering new insights into the complex relationship between language, power, and cultural identity in historical diplomatic contexts.

## Lefevere’s translation manipulation theory

Translation Manipulation Theory, proposed by Andre Lefevere in the 1990s, represents a significant paradigm shift in translation studies. It is argued that translation is essentially a form of rewriting, inherently involving manipulation of the source text to serve purposes [8]. Translation is not merely a linguistic transfer, but a complex process influenced by ideology, poetics, and patronage. This theoretical framework emphasizes how translations are produced under specific constraints, including ideological preferences, linguistic considerations, and cultural requirements [8].

It offers a comprehensive approach to understanding translation as a sociocultural phenomenon and provides valuable insights into how translations are shaped by power relations, cultural systems, and institutional constraints. As Bassnett and Lefevere noted in their collaborative work, translation manipulation theory has fundamentally changed how scholars view the relationship between source texts and their translations [9].

It has generated substantial scholarly interest and undergone significant development. Early studies primarily focused on literary translation, but recent research has expanded its application to various fields. Baker applied the manipulation theory to news translation, examining how ideological factors influence the translation of political discourse [10]. Similarly, Gonzalez-Ruiz investigated the manipulation of cultural elements in audiovisual translation, demonstrating the theory’s relevance in contemporary media studies [11]. Recent developments have also seen the integration of digital humanities approaches with manipulation theory. Wilson and Chen employed corpus-based methods to analyze patterns of manipulation in translated political texts [12], while Rodriguez explored how manipulation theory could be applied to machine translation evaluation [13].

Chinese scholars have made significant contributions to the development and application of Translation Manipulation Theory. Wang conducted a comprehensive review of the theory’s application in China, noting its relevance to the study of classical Chinese literature translation [14]. Zhang and Li extended the theory’s application to diplomatic document translation, focusing on how ideological factors influence translation choices in contemporary China-West communications [15]. Recent studies by Chinese scholars have also explored new dimensions of the theory. Liu investigated the role of translation manipulation in social media content [16], while Chen and Wang examined how manipulation theory could inform translation technology development and artificial intelligence applications in translation [17].

Despite its extensive application across various fields, there remains a significant gap in its application to the translation of historical treaties, particularly the Treaty of Nanking. While existing studies have explored diplomatic translation and its linguistic and political dimensions [10,15], no systematic research has applied Lefevere’s framework to analyze the psychological dynamics and translation strategies employed in such contexts. This study aims to address this gap by providing a novel interdisciplinary approach that integrates translation studies, psychology, and history to examine how psychological factors and power asymmetries influenced the translation process of this pivotal treaty. Specifically, it seeks to explore:

1) How translators manipulated the Chinese translation of the Treaty of Nanking to servethe interests of different parties?
2) How psychological dynamics shaped the actions of Sino-British negotiators and translators during the treaty’s negotiations?
3) How socio-historical inequalities impacted these dynamics and the resulting translation strategies?

By addressing these questions, this research not only extends the application of Translation Manipulation Theory but also contributes to a deeper understanding of the complex interplay between translation, power, and psychology in historical contexts.

## Research methodology and procedures

This study employs an interdisciplinary methodology that combines textual analysis, psychological frameworks, and historical contextualization to investigate the psychological dynamics and translation manipulation in the Treaty of Nanking, to illuminate the role of translation in reflecting and reinforcing power imbalances between the British and Chinese sides. The analysis progresses through the integration of three methodological approaches, namely, comparative textual analysis, application of psychological framework, and historical contextualization.

First, the study conducts a systematic comparative textual analysis of the Chinese and English versions of the Treaty of Nanking. This method was selected as the primary approach because it enables direct identification of linguistic disparities, rhetorical adjustments, and manipulation patterns that reveal the ideological and practical purposes underlying translation choices. As suggested by Lefevere, translation is not far from being a neutral transfer of meaning but a politically motivated process, shaped by the dominating power’s agenda [8]. The comparative textual analysis methodology follows Baker’s framework for analyzing translation in politically sensitive contexts, which emphasizes the significance of examining how the shifts in terminology, textual structure, and rhetorical devices reflect power dynamics [18]. The systematical textual comparison of the treaty reveals how British translators, led by John Robert Morrison, strategically adapted the text of the treaty to resonate with the Qing officials’ cultural expectations while ensuring the substantive terms in the English text secured British imperialist objectives.

The comparative analysis focused on three key dimensions: the structural and formatting differences between the English and Chinese versions; the terminological and semantic shifts, and the strategic additions and omissions. Primary sources examined include the original English and Chinese treaty documents, supplemented by diplomatic correspondence and official records, to provide context for the translators’ decision-making. This approach follows Venuti’s recommendation to examine both the textual products and the surrounding documentary evidence to reconstruct translation processes [19].

Secondly, this study adopts specific psychological frameworks for the analysis of the dynamics underpinning the translation processes. Three primary psychological frameworks were applied for the analysis: the power asymmetry theory developed by Keltner et al. [20], which is used to examine how power differentials influence the translators’ behavioral and linguistic choices; the cognitive dissonance theory proposed by Festinger [21], which is employed for the explanation of how translators reconciled conflicting loyalties and responsibilities; and the face negotiation theory advocated by Ting-Toomey [22], which offers insight into how cultural concepts of dignity and respect influenced translation choices. These frameworks were selected for both their demonstrated applicability to cross-cultural negotiation contexts and their ability to explain the psychological pressures experienced by both British and Chinese participants.

These frameworks were applied in this study through systematic analysis of primary source materials, including the English and Chinese versions of the treaty, diplomatic correspondence, and contemporary accounts that document the negotiators’ and translators’ attitudes, motivations, and strategic considerations. This approach follows the methodology for reconstructing historical translation contexts suggested by Chang, which highlights the importance of triangulating textual evidence with contextual documentation [23]. While acknowledging the limitations of historical psychological analysis, this approach allows for evidence-based inferences about motivations and strategies rather than speculative attribution of psychological states. For example, Keying letters and official correspondence provide direct evidence of his linguistic strategies and diplomatic considerations that informed his translation choices [5,7].

Third, the study employs historical contextualization to situate the textual and psychological analyses within the broader socio-political landscape of the early 19th century. With Wakabayashi’s methodology for historical translation studies, the study examines how prevailing diplomatic practices, cultural differences, and institutional constraints shaped the translators’ lexical and syntactic choices [24]. Primary source materials, including diplomatic archives, personal and official correspondences, and official records from both British and Chinese sides, provide the foundation for reconstructing the historical context. Secondary historiographical sources, particularly those addressing Sino-British power relations during the First Opium War period, provide additional contextual frameworks [5,25].

This approach examines specifically the institutional structures and power relations that constrained translators’ choices, the dominant diplomatic conventions in both British imperial and Qing court contexts, and the contemporary understandings of translation’s role in diplomatic processes. It follows the methodology for examining translation in colonial contexts suggested by Liu, which emphasizes the significance of understanding how broader political and cultural forces shape linguistic mediation [26].

The integration of these above-mentioned three methodological approaches sets up a comprehensive framework for understanding the multidimensional nature of translation manipulation in the translation of the Treaty of Nanking. Thorough the scrutiny of the textual discrepancies with consideration of both psychological motivations and socio-historical contexts, this research avoids oversimplified attributions of translation choices to either purely linguistic constraints or deliberate manipulation. Instead, it offers a subtle understanding of how linguistic, psychological, and historical factors interacted in this important historical text.

In brief, this methodology with integration of textual analysis, psychological frameworks, and historical contextualization, is aligned with recent developments in translation studies, which advocate for integrated approaches that combine textual, psychological, and contextual analyses. This study mitigates these limitations through rigorous triangulation of multiple primary sources and carful distinction between evidence-based inferences and speculative interpretation, while acknowledging the methodological challenges of historical research, particularly regarding psychological dynamics.

## Findings: Translation manipulation in Treaty of Nanking

The English – Chinese translation of the Treaty of Nanking demonstrates significant manipulation in the format, titles and honorifics and content during the translation process. These manipulations reflect the unequal power dynamics between Britain and China during the treaty’s negotiation and ratification. This section analyzes the translation manipulation in two dimensions: formal adjustments, which involve formal and structural changes of the Chinese version to align with Qing diplomatic conventions, and substantive changes, which include omissions, additions, and adaptations that altered the meaning of key clauses.

### Format manipulations in Chinese version

Format manipulation of the Treaty of Nanking primarily involved the format adjustments in the Chinese version to align with Qing diplomatic norms and preserve the symbolic authority of the emperor. The Chinese version of the Treaty of Nanking exhibits deliberate manipulations in textual formatting to preserve the symbolic superiority of the Qing emperor over Queen Victoria, despite China’s disadvantaged position.

**Fig 1:**
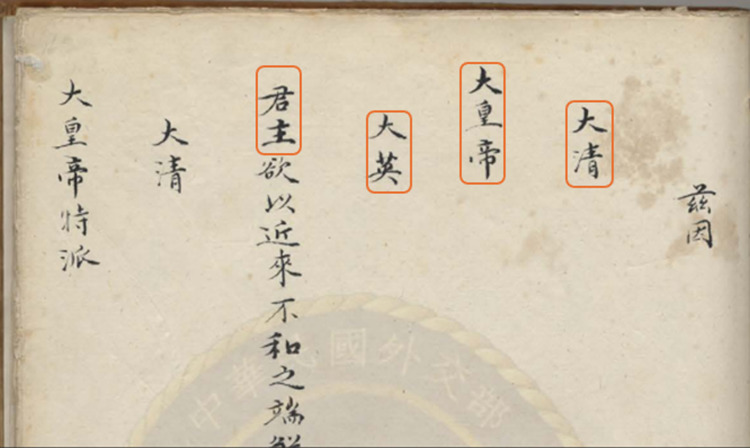
The Chinese translation of Treaty of Nanking from Taipei Palace Museum. (Source of picture: https://baike.baidu.com/item/%E5%8D%97%E4%BA%AC%E6%9D%A1%E7%BA%A6/359269).

One notable manipulation lies in the formatting of the Chinese text. Important terms such as “大皇帝” (Great Emperor) are consistently written with elevated formatting, including being placed at the top of new lines and raised by three spaces to signify respect. In contrast, “君主” (Monarch), referring to the British queen, is also written in a new line but positioned slightly lower (as shown in Fig 1).

This subtle difference, often amounting to a half-character space, is repeated throughout the document, creating a visual hierarchy that symbolically elevates the Qing emperor above the British monarch. In addition, the Chinese character “大” (great) is reserved exclusively for the Qing emperor’s title, “大清大皇帝 (the Great Emperor of the Great Qing)” and it remained in double-elevated position in the text, while it is omitted in the British monarch’s title, “大英君主(Monarch of Great Britain)”, and it was no longer written in an elevated position, and the same applied to Britain’s name: not only was the prefix “Great” removed, but it was also no longer written in an elevated position. All these further emphasized the perceived superiority of the Qing.

These manipulations reflect the Qing Dynasty’s hierarchical worldview, where the emperor was regarded as the “大国天子(Son of Heaven of a great nation)”, while the British monarch was perceived as a “夷妇 (barbarian woman)” of a lesser state. This cultural perception shaped the Chinese negotiators and translators’ approach to the Chinese translation, particularly in their efforts to maintain the emperor’s dignity.

These alterations of formatting were not without controversy. According to Yang & Gao, British negotiators was aware of the symbolic implications and protested such practices and hence demanded that they should adhere to the principle of equality in parallel documents [5]. While Qing officials, such as Qiying, conceded to British demands on substantive issues like territorial cessions, employed subtle textual strategies to assert the emperor’s preeminence, and justified these differences as stylistic or accidental to deflect the British objections.

The Chinese version of the Treaty of Nanking thus reflects the Qing’s psychological and cultural struggle to reconcile their traditional Sino-centric worldview with the realities of Western diplomatic practices. Through subtle yet deliberate textual manipulations in the translation, the Qing negotiators and translators sought to symbolically assert their emperor’s superiority, even as the treaty marked a significant loss of sovereignty. These strategies underscore the role of translator as a mediator and the role of translation as a site of ideological resistance and cultural negotiation in the face of unequal power dynamics.

### Translation of titles and honorifics

In addition to the formatting mentioned in the above section, the Chinese version of the Treaty of Nanking also demonstrates deliberate linguistic and cultural manipulations in the translation of the titles and honorifics of the Qing emperor and the British monarch. These manipulations reflect the translators’ efforts to balance the Qing’s hierarchical worldview with the Western emphasis on diplomatic equality. The choices made in translating the titles of the two monarchs illustrate subtle attempts to maintain the symbolic superiority of the Qing emperor, even within the confines of an unequal treaty.

The term “大英” (Great Britain), as a relatively recent invention, was first introduced by missionary Robert Morrison in 1831, imbuing it with a political significance that paralleled the grandeur of “大清” (Great Qing) [26]. By adopting this term, British translators sought to align Britain’s national identity with Chinese linguistic conventions, thereby accommodating the Qing’s hierarchical worldview. However, the pairing of “大英君主” with “大清大皇帝” reveals an inherent asymmetry. The term “君主” (monarch) lacks the grandeur and divine connotations of “大皇帝” (Great Emperor), which was deeply rooted in the Sino-centric worldview.

Besides, the Chinese translation of “君主” instead of “女王” (queen) for Queen Victoria was also significant. It is highlighted by Zhuang that the term “王” (king or queen) in the Qing context was typically associated with vassal states, such as Korea, Ryukyu, and Siam, which were subordinate to the Qing empire. By using “君主(monarch),” the translators aimed to convey a sense of equality with the Qing emperor. However, when placed alongside “大皇帝(Great Emperor),” the term “君主(monarch)” appears obviously less prestigious, which subtly reinforced the superior status of Qing emperor [27, p. 783].

In addition, a page-long detailed preamble containing the titles and honorifics of the Queen and other details of the treaty (as follows), is included in the English version of the treaty, which is simply absent in the Chinese version.

VICTORIA, by the Grace of God, Queen of the United Kingdom of Great Britain and Ireland, Defender of the Faith, etc, etc, etc. To All and Singular to whom these Presents shall come, Greeting! Whereas a Treaty between Us and Our Good Brother The Emperor of China, was concluded and signed, in the English and Chinese Languages, on board Our Ship the Cornwallis, at Nanking, on the Twenty-ninth day of August, in the Year of Our Lord One Thousand Eight Hundred and Forty-two, by the Plenipotentiaries of Us and of Our said Good Brother, duly and respectively authorized for that purpose; which Treaty is hereunto annexed in Original.

This preamble, as a formal and routine part of such documents, provides basic information about the treaty and outlines the treaty’s context, characterized by its religious overtones, which includes references to Queen Victoria as “by the Grace of God, Queen of the United Kingdom of Great Britain and Ireland, Defender of the Faith” [28]. Such titles emphasize the divine and sovereign authority of the British monarch. In contrast, the Qing emperor is referred to simply as the “Emperor of China” in the English text, with no additional honorifics.

Furthermore, in the English version, the Qing emperor is referred to as the “Emperor of China” and “Our Good Brother”, a customary expression used by the British when signing treaties with other nations and was deeply rooted in Christian cultural practices. However, this phrase was entirely omitted by the translators, as it conflicted with the Qing’s perception of the British monarch as a subordinate, and the Qing court and public regarded Britain as a “barbarian” nation and the British monarch as a “barbarian woman.” It was inconceivable for the Chinese emperor to refer to the British monarch as a “brother” [5, p. 186]. The Chinese version avoids glorious titles of the British monarch, which underscores the cultural and ideological tensions underlying the treaty’s translation.

Obviously, the Chinese preamble was not a direct translation of the English text but was instead adapted to address issues of interest to both parties. The English version demonstrates inequality in the titles, honorifics, and formalities for the two monarchs, while the Chinese version omits any mention of the British monarch’s titles or honorifics, nor does it include grandiose phrases for the Qing emperor. It is likely that the Qing negotiators, who did not understand English, were largely unaware of these discrepancies.

In addition, the English and Chinese texts also differ in their treatment of procedural details. The English version devotes a whole page to include comprehensive information about the treaty’s signatories, location, and date, emphasizing its legal and diplomatic rigor [29], while the Chinese version, in contrast, simplifies these details, focusing instead on the emperor’s authorization and the treaty’s practical implications. As shown below, the underlined content was all omitted. This discrepancy highlights the Qing’s prioritization of symbolic representation over procedural precision, or as it may be said that the Qing did not have much knowledge or experience of such diplomatic practices and issues.

English version: Done at Nanking and Signed and Sealed by the Plenipotentiaries on board Her Britannic Majesty’s ship Cornwallis, this twenty-ninth day of August, 1842, corresponding with the Chinese date, twenty-fourth day of the seventh month in the twenty-second Year of TAOU KWANG. (L.S.) HENRY POTTINGER, Her Majesty’s Plenipotentiary. Chinese Signatures (3). | Chinese Seal. | We, having seen and considered the Treaty aforesaid, have approved, accepted, and confirmed the same in all and every one of its Articles and Clauses, as We do by these Presents approve, accept, confirm, and ratify it for Ourselves, Our Heirs, and Successors: Engaging and Promising upon Our Royal Word, that We will sincerely and faithfully perform and observe all and singular the things which are contained and expressed in the Treaty aforesaid, and that We will never suffer the same to be violated by any one, or transgressed in any manner, as far as it lies in Our Power. For the greater Testimony and Validity of all which, we have caused the Great Seal of Our United Kingdom of Great Britain and Ireland to be affixed to these Presents, which We have signed with Our Royal Hand. Given at Our Court at Windsor Castle, the Twenty-eighth Day of December, in the Year of Our Lord One Thousand Eight Hundred and Forty-two, and in the Sixth Year of Our Reign. (Signed) VICTORIA R. The preamble mentions two Chinese negotiators only, KEYING and ELEPOO. The third signature was that of NIUKIEN, Liang Kiang Viceroy.

Chinese version: 道光二十二年七月二十四日即英国记年之一千八百四十二年八月二十九日由江宁省会行大英君主汗华船上铃关防.

A careful comparison suggests that the underlined part of the English version was all omitted in the Chinese version, retaining only the date and place when and where the treaty was signed.

As can be seen from what have been discussed above, the formatting and translation of monarchic titles and honorifics in the Treaty of Nanking exemplifies the psychological and cultural dynamics between the Qing and British translators. The British sought to establish diplomatic equality through terms like “大英君主,” while the Qing translators subtly manipulated the Chinese text to maintain the emperor’s symbolic superiority. These manipulations reveal the translators’ dual role as cultural intermediaries and ideological manipulators, navigating the tensions between traditional Sino-centric values and the realities of Western diplomatic practices.

### Substantive manipulation

Substantive manipulation in the English – Chinese translation of the Treaty of Nanking involved deliberate changes to the content of the treaty, including omissions, additions, and changes that altered the meaning of key clauses, which are highlighted below in separate tables. These manipulations reveal the translators’ active role in shaping the treaty’s narrative to save the face of Qing emperor and serve British interests while obscuring its unequal terms.

Although the Treaty of Nanking consists of only 13 articles, the translators manipulated the Chinese translation by frequently employing such methods and techniques as adaptation, addition, and omission to make technical alterations to the semantics and the content [5]. The English and Chinese versions were carefully examined and such alterations made by the translators were identified and made into the following tables for further analysis.

As shown in Table 1 above, one notable discrepancy is that “恩准 (graciously approves)” and “准(approves/grants)”, were repeatedly used as translation of “agrees” in Article II, III, V, VIII, IX in the Chinese versions. The choices of such terms as“恩准(graciously approves)” and “准(approves/grants)” in the Chinese version, which reflects an unequal relationship, to correspond to the neutral “agree” in the English text, which is an equal and neutral term, were made by the translators to emphasize the inequality of power.

**Table 1:** Changes in the English Chinese translation of the Nanking Treaty.

English	Chinese Translation	Back Translation
Her Majesty the Queen of the United Kingdom of Great Britain and Ireland, and His Majesty the Emperor of China, …	……大清大皇帝, 大英君主, ……	The Great Emperor of the Great Qing, Queen of Great Britain
ARTICLE II. … Emperor of China agrees …, … reside, … … Cities and Towns … …to see that …	……大皇帝恩准……, ……寄居…… …… 港口……, ……令……	Emperor of China graciously approves be dependent on others for shelter ports supervise/inspect
ARTICLE III. … the Emperor of China cedes, …, … in perpetuity…	三、……大皇帝准将……给予……, ……常远据守主掌。	the Emperor of China approves to bestow to defend, oversee, and control for the long term
ARTICLE IV. … imprisoned …	四、……强留……	forced to stay
ARTICLE V. … China agrees to … … agrees to pay to	五、 ……准以…… ……酌定……	approves/grants agreed and decided by both parties
ARTICLE VI. …Her Britannic Majesty…to demand and obtain redress …	六、 ……讨求伸理……,	to seek a just resolution or fairness
ARTICLE VII. … on or before the 30th of the month of June… … before the 31st of December … …stipulated…,	七、 ……六月间…… ……十二月间…… 酌定	in June; in December; agreed and decided by both parties
ARTICLE VIII. The Emperor of China agrees to release	八、 ……大皇帝准即释放。	The Great Emperor has approves/grants to release
ARTICLE IX. The Emperor of China agrees to…	九、……大皇帝…… 恩准全然免罪;……	The great emperor… graciously approves to release.

Besides “恩准,” it also employs the term “准” (to grant/permit) in multiple places during translation, resulting in similar effects. For instance, Article V of the treaty describes the trade situation in Guangzhou more elaborately in the Chinese text than in the English text. The Chinese version explicitly states: “... three million silver dollars as commercial debts, which are approved to be repaid by the Chinese officials.” In contrast, the English text conveys that the sum of three million silver dollars is paid to the British government by the Qing due to merchants’ debts to British nationals, with the “approval” of His Majesty the Emperor. In the British narrative, the Qing government paid this sum owing to merchant debts, whereas the Qing version describes it as imperial approval for officials to repay on behalf of the merchants, thereby imbuing the act with a certain “granting of favor” tone. Similarly, in Article IX, X, the word “agree” is translated as “准”, which means “granted/approved (by the emperor)”, to repeatedly highlight the Emperor’s authority.

Another point is that in Article II, the translator rendered “without molestation or restraint at the Cities and Towns of Canton, Amoy, Foochow-fu, Ningpo, and Shanghai” into “寄居大清沿海之广州、福州、厦门、宁波、上海等五处港口”, in which “cities and towns” was translated into “港口(ports)”. In the Chinese context, “港口(ports)” referred to areas around the wharfs outside the city, while “cities and towns” in the English version meant residential areas within the city walls, which are two completely different concepts. According to the English version, British civilians and their families could enjoy the same treatment as British officials, allowing them to reside in “each of the above-named cities or towns”, while the Chinese text suggests that the Qing government still did not permit ordinary British civilians to reside within the cities. This was attributable to John Robert Morrison, as he was the handler and final authority for both translations, and it is more likely for three reasons: First, Morrison, being well-versed in Chinese politics and customs, should have known the distinction between ports and cities; Second, three days after the treaty was signed, Qiying specifically explained in a diplomatic note that Britain “could only establish consulates in ports” to accommodate British merchants’ residence, showing China’s clear stance on this issue; Third, Morrison’s father, Robert Morrison, had personally experienced the hardships of foreigners being prohibited from entering cities when he first came to China [7, p. 89]. Considering these three aspects, it can be deduced that Morrison’s actions were more likely intentional. This way, he could both deceive the Chinese side while providing grounds for future demands for city entry – a “natural” course of action.

In addition, in Article III of the Treaty of Nanking, the English text uses the term “cedes,” which literally means to surrender territory or rights under military or political pressure. However, in the Chinese version, it was translated as “给与(give or bestow)”. Compared to the unequal nature implied by “cede” under power coercion, the Chinese term “给与 (give or bestow)” creates a more balanced interaction between the parties, though not as extreme as conveying imperial benevolence. The addition of the character “准” (grant/approve) before “给与(give or bestow)” further transforms the passive act into an active one, which highlights the Qing emperor’s exercise of authority. This translation choice aligns more closely with the Qing court’s initial stance on Hong Kong, which was to “graciously give or bestow” [30, p. 85]. The translator’s decision to render “cedes” as “给与(give or bestow)” represents a strategic manipulation aimed at elevating the Qing emperor’s image and creating expressions more favorable to the Qing side.

Next, in the same article, the phrase “possessed in perpetuity” was translated as “常远据守主掌 (to defend, oversee, and control for the long term)” in the Chinese version. A literal translation of “possessed in perpetuity” would have undoubtedly struck a deeper nerve with the already fragile Qing court. The term “in perpetuity” carries an absolute meaning, implying that Britain’s possession of the island after the cession would be irrevocable. In contrast, “常远据守主掌 (to defend, oversee, and control for the long term)” is a more relative term. While it still indicates that Britain will hold possession for the foreseeable future, it does not completely close the door to the possibility of its eventual return. This choice of wording could alleviate the Qing dynasty’s anxiety, from the emperor to his officials and subjects, about the question of “how to resolve this for eternity.”

In Article VI, the English version is “…Her Britannic Majesty…to demand and obtain redress …” where the underlined phrase “to demand and obtain redress”, which literally means “(commonly used in legal, political, or diplomatic contexts) to describe the process of seeking justice or remedy for a harm or violation, and then successfully achieving that remedy”, was translated into “讨求伸理”, which means “to seek a just resolution or fairness, often in response to a perceived injustice or grievance”. In the English expression, the British side condemns the harm and losses caused by the Qing Dynasty, while in the Chinese expression, the meaning is that the British side is appealing to the Emperor of the Qing Dynasty, hoping that the emperor will deliver justice on their behalf and help them seek fairness. It is evident that the translators deliberately elevated the status of the Qing emperor and maintained the symbolic superiority of the Qing emperor, even within the context of an unequal treaty.

In Article VII of the English version of the Treaty of Nanking, the payment schedule is specified with precise deadlines such as “on or before the 30th of the month of June” and “on or before the 31st of December.” However, the Chinese version adopts a more lenient tone, replacing these stronger expressions with phrases like “六月间(in June)” and “12月间 (in December).” Obviously, the tone in the Chinese version is much less aggressive compared to the English version, making it easier for the Qing emperor to accept.

Besides these changes made in the Chinese version, several additions were also made by the translators. Table 2 summaries all the additions in the Chinese version.

**Table 2:** Additions in the English Chinese translation of the Nanking Treaty.

English	Chinese Translation	Back Translation
ARTICLE III.	三、(因大英商船远路涉洋, 往往有损坏须修补者), ……	(The British merchant ships, due to their long-distance ocean voyages, often suffered damage requiring repairs.)
ARTICLE VII.	七、……(自壬寅年起至乙巳年止, 四年共交银二千一百万银元。)……	(a total sum of 2l millions will be paid in 4 years from 1842 to 1845.)
ARTICLE IV	四. ……(索出鸦片以为赎命)……	(demanding the surrender of opium as ransom)
ARTICLE X.	十、……(以便英商按例交纳);……	(... for the English merchants to pay the due amounts.)
ARTICLE XII.	十二、…… (即将驻守二处军士退出, 不复占据。)	(… then withdraw the soldiers from these two military sites and no longer occupy them.)
ARTICLE XIII.	十三、(是以另缮二册, 先由大清钦差便宜行事大臣等、大英钦奉全权公使大臣各为君上定事, 盖用关防印信, 各执一册为据, ……,要至和约者。)	(Two copies of this treaty should be separately prepared, first agreed upon by the Imperial Commissioner of the Great Qing Empire and the Plenipotentiary of Great Britain on behalf of their respective Sovereigns, and then be affixed with official seals, each party retaining one copy as evidence, with the ultimate objective of establishing and maintaining peace.)

As can be seen from the above table, in Article III of the Treaty of Nanking, the Chinese version unilaterally inserted the phrase “因大英商船远路涉洋(The British merchant ships, due to their long-distance ocean voyages, often suffered damage and need repairs.)” before the clause regarding repair requirements. By the addition of such text, the translator provided more substantial justification for the Qing court’s cession of the territory and, to some extent, enhanced the psychological acceptability of the territorial concession among the Qing emperor and his officials [5]. This deliberate textual manipulation demonstrates how translation strategies were employed to mitigate the political and psychological impact of unfavorable treaty terms on the Chinese side.

Next, in Article IV, the translators added“ 索出鸦片以为赎命 (demanding the surrender of opium as ransom)” in Article IV and “以便英商按例交纳 (for the English merchants to pay the due amounts)” in article V in the Chinese version respectively, of which there are no mentions in the English version. These expressions work in favor of the Qing government by implicitly highlighting the unjust nature of the Opium War, whereas the English version downplays this aspect entirely.

In Article VII, the sentence “自壬寅年起至乙巳年止, 四年共交银二千一百万银元” was added to the Chinese version, which literally means “a total sum of 2l millions of dollars will be paid in 4 years from 1842 to 1845.” The negotiators and translators mentioned the compensation amount here again because the British side initially demanded 30 million, but through negotiations, the Chinese negotiators and translators ultimately secured a reduction to 21 million [19, p. 27]. They considered this a significant achievement on their part.

In article XII, the sentence “即将驻守二处军士退出, 不复占据”, which literally means “then withdraw the soldiers from these two military sites and no longer occupy them” was added to the Chinese version. Here, the translators added this content to emphasize that after the compensation is paid, these places will no longer be occupied, subtly persuading the Qing emperor to pay the compensation to settle the matter peacefully. The same is true with the addition of Article XIII., which again suggests the Qing emperor finish the procedures of the treaty “with the ultimate objective of establishing and maintaining peace”.

In addition to changes and additions to the articles of the treaty, the translators also omitted some phrases or words in the Chinese version. All the omissions in the Chinese translation were collected and made into Table 3.

**Table 3:** Omissions in the English Chinese translation of the Nanking Treaty.

English
ARTICLE V. …The Government of China having the British Merchants trading at Canton to deal exclusively with certain Chinese Merchants called Hong Merchants…,
ARTICLE VI. …Her Britannic Majesty’s Plenipotentiary, …, to deduct from the said amount of Twelve Millions of Dollars,…
ARTICLE VII. shall be paid as follows: Six Millions …,
ARTICLE VIII. The Emperor of China agrees to release …,
ARTICLE XII. … at Chinhai will also be withdrawn

As illustrated in Table 3, Article V contains the phrase “having compelled” in the clause “The Government of China having compelled the British Merchants trading at Canton to deal exclusively with certain Chinese Merchants…”. This phrase was deliberately omitted in the Chinese translation. The expression “having compelled” functions as a direct denunciation of the Chinese government, attributing blame and responsibility to it. Within the hierarchical framework of the Chinese feudal system, however, such language would have been deemed unacceptable, as it was impermissible to criticize or challenge the emperor’s actions. The omission of this phrase in the Chinese version can thus be understood as a strategic decision by the translators to avoid provoking imperial displeasure or creating unnecessary conflict, aligning the text with the cultural and political sensitivities of the Qing court.

Similarly, in Article VI, the phrase “voluntarily agrees” in the English clause “Her Britannic Majesty’s Plenipotentiary voluntarily agrees…” was also omitted in the Chinese translation. This omission reflects the pervasive influence of imperial authority in Chinese feudal society, where the emperor’s willpower was regarded as supreme and absolute. In such a context, the notion of “voluntary agreement” would have been incongruous, as all actions were presumed to align with the emperor’s unquestionable authority. The translators’ decision to exclude the phrase “voluntarily agrees” served a dual purpose of reinforcing the feudal ideology of imperial supremacy on the one hand and ensuring that the omission did not significantly alter the practical meaning of the treaty provision on the other.

In Article VII of the English version of the Treaty of Nanking, the term “immediately” was used to specify the initial payment deadline, leaving no room for flexibility. However, the Chinese version simply omits it, and hence significantly reduces the sense of urgency and compulsion. Similarly, in Article VIII, concerning the release of imprisoned British subjects, the English version uses a phrase “release unconditionally”. The translators of the Chinese version deliberately omitted the term “unconditionally,” which results in a significant legal distinction between the unconditional release of prisoners and the simple act of releasing British detainees, with the former carrying far more severe implications than the latter. It is evident that the translators employed a strategy of attenuation in these instances. This approach made the Chinese phrasing appear less forceful while simultaneously serving the purposes of achieving the colonial objectives while maintaining diplomatic courtesy in dealings with the Qing dynasty. Besides, In Article XII, the term “The Military Post” in the sentence “The Military Post at Chinhai will also be withdrawn” was simple omitted in the Chinese translation. It can be inferred that the deployment of such military posts on the emperor’s land, without any question, would make the emperor unhappy. To avoid such unhappiness or the troubles that might incur, the translators intentionally omitted it.

By omitting phrases that might undermine the emperor’s authority or challenge the hierarchical structure of Qing governance, the translators effectively adapted the treaty to the sociopolitical realities of the time. Such manipulations highlight the translators’ role not merely as linguistic intermediaries but as active participants in negotiating the psychological and cultural dynamics between the two parties. As argued by Venuti, translation is inherently a form of cultural negotiation, often reflecting the power imbalances and ideological priorities of the context in which it occurs [19]. In this case, the translators sought to maintain the emperor’s absolute authority while simultaneously ensuring that the treaty’s essential terms remained enforceable.

## Discussion: Analysis of psychological dynamics

The Treaty of Nanking, signed in 1842, was not merely a product of military and political coercion. Its content, language, and translation reflect deeper psychological and cultural dimensions that influenced the decisions and strategies of both Chinese and British negotiators and translators. These dynamics manifest in the deliberate manipulation of language, strategic omission and addition of content, and the adaptation of rhetoric to meet political and cultural needs. By employing frameworks of power asymmetry, cognitive bias, emotional strategies, this section explores the psychological pressures and constraints shaping the treaty’s final form.

### Negotiators and translators of the Treaty of Nanking

A unique characteristic of the Treaty of Nanking negotiations was that the roles of negotiators and translators significantly overlapped, which created complex psychological dynamics that profoundly influenced both the negotiation process and translation outcomes. Such practice was particularly significant in the context of Sino-British relations during the 19th century, marking a departure from traditional diplomatic practices [7,31].

On the one hand, the British side maintained complete control over the translation process, with three key figures – John Robert Morrison, Karl Friedrich August Gutzlaff, and Robert Thom – serving not merely as linguistic intermediaries but as active participants in shaping the treaty’s content and presentation [3,5]. Among them, Morrison’s role was particularly significant as he functioned simultaneously as chief translator, diplomatic advisor, and de facto negotiator. This concentration of roles created a unique power dynamic where translation became inseparable from diplomatic strategy [3,5,7].

The psychological implications of this role convergence were profound. As chief translator, Morrison faced the complex task of balancing multiple, sometimes conflicting responsibilities: maintaining diplomatic protocol, serving British imperial interests, and preserving the appearance of fair negotiation. It is repeatedly addressed that this psychological tension was evident in his translation choices, which often reflected strategic diplomatic considerations rather than purely linguistic concerns [5].

On the other hand, the situation was markedly different on the Chinese side. The Qing negotiation team, led by Keying and Elepoo, lacked any officials with English language proficiency or translation expertise. According to historical records, they were forced to rely entirely on British interpreters for all communication and document translation [1,3,5,6]. This dependency created significant psychological pressure on the Chinese negotiators, who had to make critical decisions without direct access to or understanding of the English versions of diplomatic communications.

The power asymmetry in translation control had significant implications for the dynamics of negotiation. British translators could strategically manage both the initial drafting and final translation processes, while Chinese officials were limited to reviewing only the Chinese versions of documents. This arrangement created a “controlled mediation” [15] environment, where British translators could subtly influence treaty terms through strategic translation choices while maintaining the appearance of diplomatic equality.

The psychological impact of this arrangement was particularly evident in the treaty’s final form. Historical records indicate that British translators often employed “strategic ambiguity” [16] in their translations, carefully balancing explicit treaty requirements with more subtle linguistic choices that could serve future British interests while maintaining acceptable diplomatic form. This approach reflected the complex psychological calculations involved in serving as both translator and negotiator.

This unique convergence of roles had lasting implications for both the immediate treaty outcomes and broader Sino-British relations. The translation process became not just a means of communication but a tool for exercising diplomatic power, with translators serving as key agents in shaping the relationship between the Qing Dynasty and the British Empire [4].

### Psychological dynamics of British negotiators and translators

The British side approached the treaty negotiations with a complex set of psychological motivations stemming from their dual roles. Their psychological dynamics were characterized by three main aspects: strategic manipulation, cultural superiority, linguistic advantage and personal interest [32]. These psychological aspects were manifested through specific cognitive and behavioral patterns that influenced their translation decisions throughout the treaty negotiation process.

First, as negotiators, they exhibited a clear understanding of their military and diplomatic superiority, which manifested in their translation choices. This cognitive awareness of power asymmetry, as conceptualized by Chen, led to what Thompson terms “strategic confidence” in diplomatic interactions [32,33]. This awareness led to deliberate manipulation of certain terms to reinforce British dominance while maintaining an appearance of equality. For instance, the comparatively rigid form and careful selection of diplomatic terminology in the Chinese version often masked the treaty’s coercive nature while preserving its legal force. Letters and diplomatic correspondence from the period provide evidence of this calculated approach, with Morrison writing to Pottinger that “certain adjustments to the Chinese text will make the terms more palatable without sacrificing our essential demands” [34].

Second, their role as translators provided them with significant psychological leverage. Morrison, in particular, used his linguistic expertise to craft the Chinese translation to serve British interests while remaining technically accurate. This dynamic reflects what Baker identifies as “narrative framing” in translation, where linguistic choices are made to position the translator’s side favorably. This dual responsibility created the “strategic ambiguity,” [35] wherein specific linguistic choices were deliberately employed to modify meaning while maintaining plausible deniability of manipulation. For example, the translators’ change of “on or before the 30th of the month of June” and “on or before the 31st of December” into “六月间 (in June)” and “12月间 (in December)” is not merely a linguistic simplification but a calculated tactic to create temporal flexibility that could later benefit British side. While some such changes might be attributed to structural differences between English and Chinese temporal expressions, it is argued that the consistent pattern of these modifications suggests deliberate strategic intent rather than mere linguistic necessity [36].

Third, archival records reveal that the British translators experienced psychological tension in balancing diplomatic objectives with cultural sensitivity [37]. As Wang’s analysis of treaty port communications demonstrates, British translators were acutely aware of Chinese cultural sensitivities and deliberately calibrated their translations to avoid unnecessary provocations while preserving British advantages [31]. The psychological pressure of maintaining British imperial interests while ensuring the treaty’s acceptance by the Qing court led to sophisticated manipulation strategies. The British negotiator-translators often employed “cultural bridging” techniques [38]. Under the influence of the “Sinocentric worldview,” or the concept of “distinguishing between civilization and barbarians”, the Qing Empire consistently adopted a condescending attitude toward foreign envoys, which was directly reflected in its diplomatic etiquette and rhetoric [5, pp. 189–190]. This created what Liu describes as a “cognitive challenge” for British translators who needed to operate within an unfamiliar cultural framework while advancing their objectives [38]. Although the Treaty of Nanking was the first unequal treaty in modern Chinese history, it still retained the previous discourse system of imperial supremacy, which can be seen throughout the Chinese version of the treaty. The translators adapted the Chinese discourse system for the Western diplomatic language while ensuring the preservation of British advantages.

Last, the English translator, John Robert Morrison, not only represented British national interests but also intertwined personal agendas and legacies in the negotiation and translation of the Treaty of Nanking. As chief translator, Morrison influenced critical clauses with far-reaching consequences, notably creating discrepancies between Chinese and English versions regarding residency rights. The English text ambiguously granted British subjects liberties beyond what the Chinese side had agreed upon, particularly the contentious “right to reside in cities,” which later justified British demands in protracted “anti-entry-to-city” conflicts. Morrison’s actions were unlikely mere linguistic limitations or cultural misunderstandings given his familiarity with Chinese political nuances. Personal motives likely influenced his translation choices, as his father, Robert Morrison, had endured struggles under Qing restrictions against foreigners residing within cities—potentially fueling a desire to address historical injustices. Moreover, Morrison exploited Qing officials’ inability to verify the English text due to their lack of English fluency and dependence on Chinese versions. This intentional introduction of disparities between treaty versions exemplifies how translation was manipulated to serve both imperial and personal interests, embedding Morrison’s individual will into an already unequal negotiation process [7,39].

In brief, when Morrison and others translated the Treaty of Nanking from English into Chinese, they made various adaptations in ways that would make the Chinese version more acceptable to the Qing court and even actively downplaying the British stance. These adaptations can be categorized along a spectrum from necessary linguistic accommodations (such as honorific terms required by Chinese diplomatic convention) to deliberate ideological manipulations (such as strategic omissions of terms that might provoke resistance) [23]. The purpose of their manipulation of the translation may be to preserve the dignity of the Qing government and avoid escalating conflicts, and hence to facilitate the swift signing of the treaty by adopting more flexible wording or portraying the treaty as a voluntary act of the Qing emperor, which would thereby frame the British aggression in a more favorable light. Comparative analyses with other contemporary treaty translations, such as the Treaty of Wangxia (1844) between China and the United States, reveal similar patterns of psychological management through translation, suggesting these were not isolated practices but part of a broader Western approach to diplomatic translation with China [40]. Such manipulation resulted in a translation that simultaneously preserved the Qing government’s face and disguised the coercive nature of the unequal treaty imposed by Britain, creating an illusion of amicable harmony. However, from a translation perspective, such manipulative practices deviate from the principles of translation ethics, which compromise the precision and objectivity required in legal document translation and fail to uphold the rigor and neutrality essential to this genre.

### Psychological dynamics of Chinese negotiators and translators

The Chinese side faced more complex psychological challenges due to their relatively weaker position and cultural constraints. Unlike their British counterparts, Chinese negotiators operated within a framework of what can be termed “negotiation under duress” by Zhou & Wang, in which military defeat created significant psychological constraints that directly influenced their translation and negotiation strategies [41]. Several key psychological factors were identified to influence their approach to negotiation and translation.

Primarily, the Chinese translators and negotiators operated under significant psychological stress due to their military defeat and the need to preserve imperial dignity. This manifested in what Chang terms “defensive translation strategies,” [23] where Chinese negotiator-translators attempted to maintain the Chinese traditional diplomatic language while accommodating British demands. These strategies represent a form of “psychological self-protection” [42], where linguistic choices serve to mitigate perceived threats to identity and status. Archival materials from the Qing court reveal explicit concerns about maintaining “天朝体面” (celestial empire’s dignity) throughout the negotiation process, with officials instructed to preserve rhetorical sovereignty even while conceding substantive demands [43].

Their translation choices often reflected attempts to minimize the appearance of submission while accepting the reality of their position. For example, the addition of the phrase “因大英商船远路涉洋(The British merchant ships, due to their long-distance ocean voyages, often suffered damage and need repairs) in Article III serves as what Wang identifies as a “causal reframing,” providing a pragmatic rationale for concessions that would otherwise appear as purely unilateral submissions to foreign demands [44]. Similarly, the omission of “immediately” and “unconditionally” in article VII and VIII of the Chinese versions represent a strategic employment of what psychologists’ term “linguistic distancing” [45], creating psychological space between the directive language of the English text and its Chinese rendering.

Furthermore, the Chinese translators and negotiators experienced what modern scholars call “cognitive dissonance” in their dual roles. This psychological state, characterized by mental discomfort arising from contradictory beliefs or values, manifested in specific translation choices that attempted to reconcile incompatible imperatives [21]. They had to balance their responsibility to protect Qing interests with the practical necessity of accepting unfavorable terms. Primary documents indicate this internal conflict directly affected negotiator psychology, with Keying’s memorials to the emperor revealing his struggle to justify diplomatic compromises to a court still operating within traditional tributary frameworks [46].

This internal conflict influenced their translation choices, particularly in the articles dealing with sovereignty and territorial concessions [32]. For instance, the Chinese translation of Article III’s provisions regarding British trade rights employs deliberate vagueness in describing the scope of these rights, reflecting what Pan terms “strategic ambiguity through lexical choices.” [47] These translation decisions reveal not merely linguistic accommodation, but psychological coping mechanisms deployed to manage the dissonance between preserving imperial sovereignty and accepting subordinate status in the treaty relationship.

What’s more, their psychological state was further complicated by their limited knowledge of Western diplomatic conventions and legal concepts. This knowledge gap created the “translation anxiety,” [48] where uncertainty about Western terms’ implications led to cautious and sometimes ambiguous translations of crucial provisions. This anxiety was exacerbated by the absence of standardized Chinese equivalents for Western legal concepts, creating what cognitive psychologists’ term “conceptual uncertainty” [49]. Historical evidence suggests Chinese negotiators were acutely aware of this disadvantage, with court documents from the period expressing concerns that unfamiliar terminology might conceal additional obligations or restrictions [43].

Compounding these challenges was a deeper psychological struggle related to cultural identity and imperial self-perception. The Chinese negotiators operated within what Liu terms the “conceptual framework of centrality,” where China’s position as the “Middle Kingdom” formed a core component of diplomatic self-understanding [50]. Archival materials demonstrate that even in military defeat, Chinese officials maintained psychological attachment to traditional conceptions of hierarchical international relations, resulting in translation choices that preserved formal aspects of this worldview while conceding substantive authority [51].

This psychological commitment to cultural continuity explains why, even when accepting humiliating treaty terms, Chinese translations consistently maintained imperial honorifics and traditional diplomatic phraseology. As Wong notes, these linguistic choices were not merely stylistic but represented “psychological anchoring” to established identity frameworks during a period of profound geopolitical disruption [37]. Such translation decisions provided psychological continuity amid political discontinuity, allowing negotiators to maintain cognitive coherence while engaging with fundamentally altered power dynamics.

In short, the psychological dynamics of Chinese negotiators and translators thus reveal translation not merely as linguistic mediation but as a complex process of psychological negotiation. Facing military defeat, knowledge asymmetry, and cultural dissonance, Chinese officials employed translation strategies that simultaneously acknowledged new realities while preserving essential elements of traditional diplomatic self-conception. These strategies—including defensive linguistic choices, strategic ambiguities, and cultural reframing—represent sophisticated psychological responses to the complex pressures of negotiating China’s first “unequal treaty,” revealing translation as both a site of power contestation and a mechanism for psychological adaptation to geopolitical transformation.

## Conclusion

This study has examined the complex interplay of translation manipulation and psychological dynamics in the Treaty of Nanking (1842), a pivotal document that marked a watershed moment in modern Chinese history and Sino-British relations. Through an interdisciplinary approach combining textual analysis, psychological frameworks, and historical contextualization, this research has revealed how translation served as both an instrument and a reflection of power relations in colonial encounters.

The analysis demonstrates that translation manipulation manifested in both formal and substantive dimensions. Formal manipulations, including strategic formatting and honorific language choices, were employed to preserve the appearance of Qing sovereignty while masking the treaty’s coercive nature. Substantive manipulations, encompassing deliberate omissions, additions, and semantic shifts, served to obscure the treaty’s inherent inequalities while ensuring its acceptance by the Qing court. These manipulative strategies reflect the complex role of translators as cultural mediators operating within asymmetrical power relations.

The psychological dynamics underlying the translation process proved particularly significant. British negotiator-translators, operating from a position of military and diplomatic superiority, employed sophisticated manipulation strategies while maintaining an appearance of diplomatic equality. Their Chinese counterparts faced more complex psychological pressures, struggling to reconcile their military defeat with the need to preserve imperial dignity. This cognitive dissonance manifested in defensive translation strategies and careful linguistic choices that attempted to minimize the appearance of submission while accepting unfavorable terms.

The findings of this research contribute significantly to our understanding of translation as a site of ideological contestation and cultural negotiation. The Treaty of Nanking case demonstrates how translation can serve as a mechanism for consolidating colonial power while simultaneously providing space for subtle resistance through linguistic and cultural adaptation. The analysis reveals that translation manipulation in diplomatic contexts extends beyond mere linguistic transfer, encompassing complex psychological, cultural, and political dimensions.

These findings have important implications for translation studies, particularly in understanding the role of translators in historical power relations. The study demonstrates how translators, operating under various psychological pressures and political constraints, actively shape diplomatic outcomes through their linguistic choices. This challenges traditional notions of translator neutrality and highlights the need for more nuanced approaches to studying translation in colonial and post-colonial contexts.

In conclusion, this research advances our understanding of the intricate relationship between translation, power, and psychology in diplomatic contexts. By illuminating the complex dynamics at play in the Treaty of Nanking’s translation, it contributes to broader theoretical discussions about translation manipulation, cultural mediation, and the role of translators in shaping historical narratives. Future research might productively explore how these dynamics continue to influence contemporary diplomatic translation practices and cross-cultural negotiations.
